# Small RNAs as Fundamental Players in the Transference of Information During Bacterial Infectious Diseases

**DOI:** 10.3389/fmolb.2020.00101

**Published:** 2020-06-16

**Authors:** Juan José González Plaza

**Affiliations:** Faculty of Forestry and Wood Sciences, Czech University of Life Sciences Prague, Prague, Czechia

**Keywords:** small RNA, bacteria, infectious disease, information transfer, host-pathogen interaction

## Abstract

Communication shapes life on Earth. Transference of information has played a paramount role on the evolution of all living or extinct organisms since the appearance of life. Success or failure in this process will determine the prevalence or disappearance of a certain set of genes, the basis of Darwinian paradigm. Among different molecules used for transmission or reception of information, RNA plays a key role. For instance, the early precursors of life were information molecules based in primitive RNA forms. A growing field of research has focused on the contribution of small non-coding RNA forms due to its role on infectious diseases. These are short RNA species that carry out regulatory tasks in *cis* or *trans*. Small RNAs have shown their relevance in fine tuning the expression and activity of important regulators of essential genes for bacteria. Regulation of targets occurs through a plethora of mechanisms, including mRNA stabilization/destabilization, driving target mRNAs to degradation, or direct binding to regulatory proteins. Different studies have been conducted during the interplay of pathogenic bacteria with several hosts, including humans, animals, or plants. The sRNAs help the invader to quickly adapt to the change in environmental conditions when it enters in the host, or passes to a free state. The adaptation is achieved by direct targeting of the pathogen genes, or subversion of the host immune system. Pathogens trigger also an immune response in the host, which has been shown as well to be regulated by a wide range of sRNAs. This review focuses on the most recent host-pathogen interaction studies during bacterial infectious diseases, providing the perspective of the pathogen.

## Introduction

“*Dizese calétura epidemia porque es común a muchos: perniciosa, porque mata a muchos quanto es de su parte, por tener mucha actividad de calor proveniente (como despues diremos) de vn podrecimiento extremo*.”

“*It is called an epidemic fever because it is common to many: pernicious, because it kills many on its part, due to a lot of heat activity originating (as we will say later) from extreme rotting*. de Viana (1637); plague that heavily struck the port city of Málaga.”

A continuous flow of myriads of energy and matter from atomic to beyond gravitational level shapes life on Earth. Information, its structure and movement across hierarchies, has played a paramount role on the evolution of all living or extinct organisms since the appearance of life. For instance, the early precursors of life were information molecules based in primitive RNA forms (Higgs and Lehman, 2015; Taylor, 2016). The fact that all biological species live in networks, force them to interact with other living beings and the environment (Bermúdez-Barrientos et al., 2020). Interactions, different forms of sociality, transference or reception of information, determine how species perform through evolutionary history. Communication in biology or biocommunication reaches far beyond the human concept of language (Witzany, 2010), and includes a wide variety of forms: sexual deception (color display in orchids to attract pollinators) (Streinzer et al., 2009), vibroacoustic or chemical alarm signaling (alarm pheromones in termites) (Cristaldo et al., 2016), or camouflage (How et al., 2017). Additionally, it encompass hostile communication forms, such as signals to attack among the microbiome (antibiotic production for elimination of competitive bacteria) (Fischbach, 2009; Romero et al., 2011), or counterfeit the defenses of others (fungal RNAs suppressing plant defenses) (Weiberg et al., 2013). Success or failure in the transmission of these signals dramatically affects the prevalence or disappearance of a given set of genes, the basis of Darwinian paradigm (Langdon, 2016).

Infectious diseases are biological examples of competition between species for the same metabolic resources (Herrera and Nunn, 2019), relationships that have been shaped through evolution. In essence, those are evolutionary arms races between hosts and their infectious agents (Ingle et al., 2006; Kuijl and Neefjes, 2009). An infectious disease is a malady caused by a pathogenic organism, including bacteria, fungi, parasites, or viruses (Krämer et al., 2009). The process of infection comprises the change from an outer environment, to another one inside of the host, where the conditions are hostile due to the presence of immune systems. In some animal hosts, the recognition of the pathogen could unchain a series of events leading to an inflammatory response, which represent a plethora of environmental stresses for the bacteria.

Hosts have developed sophisticated mechanisms to sense invaders, and to react against them. Diving at a very deep molecular level one of the first barriers of defense to trigger innate immune responses, is the recognition of pathogen-associated molecular patterns (PAMPs) that will further activate toll-like receptors (TLRs) pathways (Ausubel, 2005; Moresco et al., 2011). Bacteria and archaea have developed during evolution primitive adaptive mechanisms to identify “pathogens” by identification and restriction of foreign genetic material through CRISPR-CAS systems (Hille et al., 2018; Ratner et al., 2019).

The key for the infection success is a quick response and efficient adaptation to a changing hostile environment within the host (Sauder and Kendall, 2018), the translation of cues from the extracellular domain into triggering a set of instructions aimed for the survival of the pathogen. Different molecules play a role in the transference of information, for example during the formation of biofilms, sub-inhibitory concentrations of antibiotics have been proposed to serve as carriers of information between bacteria (Romero et al., 2011). Among different molecules, RNA has achieved a very important role as an information mediator, because it is the molecular link between genome (DNA) and phenotype (proteins or metabolites). It can be quickly recruited during biotic or abiotic stresses. The average life of RNA molecules is short, because the response has been *tuned* to serve for the synthesis of proteins and to be degraded once they are not needed anymore (Gilbertson et al., 2018).

Small regulatory RNAs (sRNAs) are a subset of RNA molecules that are involved in several mechanisms that aid the pathogen in adaptation, counterfeiting, or suppressing the host immune system (González Plaza, 2018), side to side to other molecules participating in this complex process. The sRNAs are transcribed from the genome, but do not follow the canonical path toward protein translation (Waters and Storz, 2009).

One reason for the involvement sRNAs at infection processes, is the flexibility to target a number of genes or transcription factors, leading to continuous ranges of expression and responses to fluctuating environmental stresses, instead of an abrupt triggering or shutting down of the expression. In that regard, Silva and collaborators have shown that SraL sRNA is responsible for regulating the expression of the transcription termination protein Rho (Silva et al., 2019). This protein is essential for the transcription balance in *Bacillus subtilis*, and its impairment affects negatively cell motility, biofilm formation, and sporulation (Bidnenko et al., 2017). Thus, the case of SraL and Rho illustrates a complex system of “regulation of regulators.” However, it could be argued that many proteins can carry out similar regulatory roles. Among other reasons, it is probably the faster response of RNAs what has given them a key role during infection, because they do not require translation and it represents a lower energy consumption for the cell. Modulation of the transcriptomic levels allows for a faster response to environmental changes (Sheehan and Caswell, 2018), because it can help to correct or modulate the mRNA levels of many genes before the protein is translated, and ultimately modulate the phenotype according to external fluctuations.

The current review article presents the advances in the field regarding the involvement of sRNAs during bacterial infections, highlighting the latest contributions in the 2 years since my previous review (González Plaza, 2018). The field has expanded broadly, and the number of contributions points toward a future increase in the number of research efforts. The current review aims as well to broaden the scope from diseases affecting humans to other species. It will first cover the type of existing sRNAs and their mode of action, the molecular behavior of sRNAs from the pathogen perspective, and modulation of molecular processes when facing host immune systems. Lastly, different type of sRNAs during several infection processes in a number of species, ranging from plants to animals.

## Types of sRnas

The subset of small regulatory RNAs, termed in literature as sRNAs, are a group of primarily non-coding RNA forms (Waters and Storz, 2009) often ranging from 20 to 200 nucleotides (nt) in length, even reaching up to 500 nt (Carrier et al., 2018a). They carry key roles regulating expression levels in a wide range of prokaryotic or eukaryotic genes (Waters and Storz, 2009; Brant and Budak, 2018; Carrier et al., 2018b). Their targets include important genes that are relevant either for the infection process or for the defense of the host organism (Guo et al., 2019). A notable feature is their reported participation in *trans*-kingdom communication (Benbow et al., 2018; Zeng et al., 2019; Bermúdez-Barrientos et al., 2020). Attending to their origin, they can be divided in prokaryotic and eukaryotic sRNAs.

### Prokaryotic sRNAs

**FIGURE 1:**
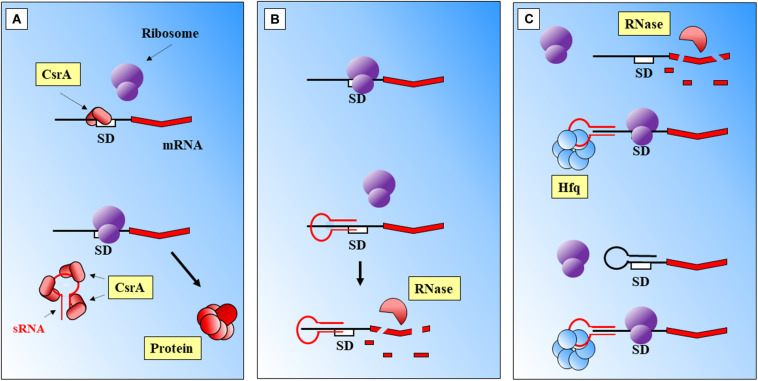
Main modes of action of prokaryotic sRNAs. **(A)** sRNA binding to a CsrA regulatory protein frees the SD region. **(B)** Direct sRNA-mRNA binding. The shown sRNA competes with the ribosome, which cannot bind to the SD site. The mRNA is further degraded. **(C)** Protein mediation. Hfq-sRNA bind to the mRNA and stabilize it, and degradation is prevented. The complex protein-sRNA can unfold secondary structures in the 5′ UTR which will prevent ribosome binding. SD, Shine-Dalgarno. Black thick arrows indicate flow of a biological process, e.g., protein translation. References: Massé et al., 2003; Waters and Storz, 2009; Faucher and Shuman, 2011; Chakravarty and Massé, 2019.
(ii) Direct sRNA-mRNA interaction (Figure 1B). Trans acting sRNAs bind to their targets with partial complementarity (Caldelari et al., 2013). There are different types of interaction, for instance the destabilization of the mRNA by pairing to upstream locations from RBS (Ross et al., 2019) or by interference with the 5′ UTR (Rübsam et al., 2018). Binding of the sRNA can also mask the ribosomal binding site (RBS), consequently the ribosome cannot bind and the translation of the gene is attenuated (Kiekens et al., 2018). The complex mRNA-sRNA can be subject of degradation by RNaseE (Lalaouna et al., 2013).
(iii) Protein mediation (Figure 1C). RNA-binding proteins (RBP) can mediate the regulatory activities of sRNAs, such as Hfq (Beisel and Storz, 2010) or ProQ (Smirnov et al., 2016). The Hfq chaperone mediates interactions between sRNAs and their targets helping to improve base-pair recognition (Massé et al., 2003; Lenz et al., 2004; Holmqvist and Vogel, 2018; Santiago-Frangos et al., 2019). This protein binds to the regulatory RNAs, helps to stabilize them, and leads the base pairing with the targets (Hu et al., 2018; Han et al., 2019). Hoekzema et al. (2019) have suggested an additional mechanism, where the action of Hfq will unfold a hairpin in the targeted mRNA. This activity would create a temporary local structure that facilitates the access of the sRNA. There are other microorganisms where Hfq protein is lacking and FinO, ProQ or RocC carry out its broad regulatory roles: sRNA protection from degradation, alteration of RNA structures to facilitate annealing, stabilization of the sRNA-mRNA complex, or modulation of ribosome binding after the complex is formed, and regulation of RNA degradation (Olejniczak and Storz, 2017).

Prokaryotic regulatory RNAs can be classified in three main groups: (i) elements present in the 5′ untranslated regions (UTR), (ii) those acting in *cis* and termed anti-sense RNAs (Lejars et al., 2019), and (iii) those acting in *trans* that are expressed from other genomic regions than their targets (Chakravarty and Massé, 2019). The third type can be originated at intergenic regions, but also at 5′ or 3′ UTR regions, and are usually termed as sRNAs (Carrier et al., 2018a; Chakravarty and Massé, 2019). The *trans*-acting sRNAs are the main focus of the current article. The regulation occurs according to several mechanisms, which have been thoroughly reviewed by Carrier, Lalaouna, and Massé (Carrier et al., 2018a). Briefly:

(i) Binding to a regulatory protein. For example binding of sRNAs to the regulatory CsrA protein, that cannot occlude the Shine Dalgarno (SD) sequence of its target (Romeo and Babitzke, 2018; Figure 1A).

### Eukaryotic sRNAs

In eukaryotes the description of the first types of sRNAs dates back to 1990 with studies reporting silencing of gene expression in petunia plants (Napoli et al., 1990; van der Krol et al., 1990), and later in 1993 in the nematode *Caenorhabditis elegans* (Lee et al., 1993; Wightman et al., 1993).

The main types of described sRNAs range from 20 to 30 nt, and carry out silencing functions by mediation of Argonaute family proteins (Kim et al., 2009). These sRNAs were firstly divided in three classes: microRNAs (miRNAs), small interfering RNAs (siRNAs), and PIWI-interacting RNAs (piRNAs) (Grimson et al., 2008; Okamura and Lai, 2008; Islam et al., 2018). Long-non-coding RNAs (lncRNAs) are a new group of described regulatory molecules over 200 nt (Agliano et al., 2019).

In the case of miRNAs it has been reported that they can control up to 60% of the human transcriptome, therefore, their involvement in the response to infectious diseases is not surprising (Aguilar et al., 2019).

## sRNAs Regulate Key Processes for the Establishment of Infection

Establishment of infection requires a prompt adaptation effort from a pathogenic perspective, in order to proliferate within the host. There are different type of environmental challenges faced by bacteria when entering the host, including different immune barriers to infection (Chakravarty and Massé, 2019). Regulation of transcription aids to adapt quickly to the newly encountered hostile conditions: changes in nutrient availability, pH, temperature, or presence of antimicrobials among other variables. Together, those force bacteria to behave differently during the infective process in comparison with the free form state. It is during these conditions when the diverse toolkit of RNA regulatory activities greatly help for survival. An overview of the different stresses that bacteria must adapt to when entering a host, and the role of sRNAs, have been summarized in Table 1.

**TABLE 1:** Adaptation to environmental stresses mediated by sRNAs.

**Category**	**Biological Process or Host Barrier**	**Type of stimulation**	**Organism**	**Infectious disease**	**sRNA**	**Mechanism of sRNA action**	**Physiological effect**	**Potential value if present in a pathogen**	**References**
Regulation of biological processes	Temperature response	Extreme heat stress	*Pseudo- alteromonas fuliginea* BSW20308	N/A	4 known sRNAs 15 novel sRNAs	Not described	Regulation of genes for adaptation to challenge, e.g., scavenging ROS, oxidation of toxic aldehydes, or antioxidant enzymes.	Adaptation to pyrexia (organisms presenting it), sensing of host temperature	Liao et al., 2019
Regulation of biological processes	Temperature response	Temperature	*Borrellia burgdorferi*	Lyme disease	>1,000	Not described	Regulation of genes involved in metabolism, cell cycle, or infection (among others)	Identification of the molecular program to trigger according to environment	Popitsch et al., 2017
Regulation of biological processes	Stringent response	Stringent response	*Borrellia burgdorferi*	Lyme disease	1/3 of sRNome regulated	Rel_Bbu_ combines enzymatic functions of RelA and SpoT.	Regulation of virulence and metabolism upon stringent response	Adaptation to host/vector/free state	Drecktrah et al., 2018
Regulation of biological processes	QS and Biofilm	Quorum-sensing response	*Pseudomonas aeruginosa*	Opportunistic infection	RhlS (+)	Binds to the 5′ UTR *rhlI* mRNA and stabilizes it, Hfq dependent	Leading to production of C4-HSL	Activation of biofilm genes according to the state of infection	Thomason et al., 2019
Regulation of biological processes	QS and Biofilm	N/A	*Pseudomonas aeruginosa*	Opportunistic infection	P27 (−)	P27 binds to the 5′ UTR *rhlI*, inhibits translation. Hfq dependent	Leading to repression of C4-HSL	Deactivation of biofilm genes according to the state of infection	Chen et al., 2019
Regulation of biological processes	QS and Biofilm	N/A	*Pseudomonas aeruginosa*	Opportunistic infection	RsmV (−)	Targets and binds RsmA and RsmF, also has redundancy of targets with known regulators	Repression of regulators involved in activating/deactivating acute/chronic infection related genes	Switching between infective lifestyles	Janssen et al., 2018b
Regulation of biological processes	QS and Biofilm	High-cell density (biofilm) Presence of membrane stressors	*Burkholderia cenopacia*	Opportunistic infection	ncS35 (−)	Potential binding to the mRNA inhibiting translation	Slows-down growth, restricts division	Triggering of infection related genes when pathogen finds the right environment	Kiekens et al., 2018
Regulation of biological processes	QS and Biofilm	N/A	*Pseudomonas aeruginosa*	Opportunistic infection	PhrD (+)	Positive regulator of RhlR by messenger stabilization (Hfq mediated)	Stabilization of *rhlR* messenger	Regulation of biofilm formation by modulation of a key regulator	Malgaonkar and Nair, 2019
Regulation of biological processes	Virulence	Environmental stress	*Pseudomonas aeruginosa*	Opportunistic infection	ReaL (−)	RpoS controls virulence factors, regulated (−) by ReaL (Hfq dependent base-pairing mechanism)	Wide downstream effects, since it regulates *rpoS* mRNA	Fine tuning of virulence factors	Thi Bach Nguyen et al., 2018
Host barriers to infectious diseases	Acid pH	pH, antimicrobials	*Escherichia coli*	Opportunistic / Enterohemorrhagic (if *Escherichia coli* O157:H7) infection	RydC (+) ArrS (+) CpxQ (−)	CpxQ-HfQ bind to mRNA, facilitate access to RNase cleavage site. RydC-HfQ and ArrS opposite effect	Modification of cell membrane versus several stresses. The enzyme transcripts (cyclopropane fatty acid synthase) stabilized and protected from RNAse E	Overcoming one of the first barriers to infection, in order to access the lower gastrointestinal tract	Bianco et al., 2019
Host barriers to infectious diseases	Inflammation	Oxidative burst	*Staphylococcus aureus*	Opportunistic infection. Severe respiratory disorders	RsaC (−)	Binding to the RBS of the gene s*odA* (protection against ROS species).	Targeted gene repression allows transcription of SodM (protection vs. ROS, uses iron as cofactor).	Maintenance of ROS protection when a cofactor is depleted by using different metallic ion.	Lalaouna et al., 2019
Host barriers to infectious diseases	Nutritional immunity	Iron starvation	*Pseudomonas aeruginosa*	Opportunistic infection.	PrrF1 (−) PrrF2 (−)	Inhibition of *antR* translation via Hfq and binding to the SD	Transcribed upon iron starvation, modulate synthesis of proteins containing limiting elements. Modulation of biofilm and virulence via targeting anthranilate degradation pathway.	Avoids synthesis of unnecessary iron-containing proteins when this compound is limited.	Djapgne et al., 2018
Host barriers to infectious diseases	Nutritional immunity	Environmental stresses related with iron withholding and nutrient starvation	*Escherichia coli* W3100	Opportunistic / Enterohemorrhagic (if *Escherichia coli* O157:H7) infection	RyhB (−)	Binds target mRNA via Hfq, allows recognition by degradasome.	Transcribed upon iron starvation, modulate synthesis of proteins containing limiting elements.	Described (previously) to avoid synthesis of iron containing proteins under iron limitation; redirection of metabolic fluxes	Lyu Y et al., 2019
Host barriers to infectious diseases	Nutritional immunity	Nutrient starvation	*Salmonella enterica* serovar Typhimurium	Diarrheal disease / Typhoid fever	STnc1740 (−) RssR (+)	RssR was suggested to bind to the 5′ UTR of *reiD*	Utilization of myo-inositol as carbon source	Redirection of metabolism, growth regardless of host nutritional starvation response	Kröger et al., 2018
Host barriers to infectious diseases	Nutritional immunity	N/A	*Vibrio cholerae*	Cholera disease	MtlS (−)	*Cis*-antisense complementation	Regulation not directly caused by the environmental cue, but target mRNA levels	Regulation of metabolic resources during host nutritional starvation response	Zhang and Liu, 2019

*This table contains a list of different adaptations to environmental stresses, either described in environmental bacteria, in characterization studies of pathogen models in the laboratory, or in studies assessing the host-pathogen interaction. Many of these stresses can be encountered by bacteria during infection. The type of small non-coding regulatory RNAs involved. +, positive regulation; −, negative regulation; C4-HSL, N-butanoyl-homoserine lactone; QS, Quorum Sensing; ROS, reactive oxygen species; UTR, untranslated region.*

The range of adaptations mediated by sRNAs have been grouped in two main related categories: (i) regulation of key bacterial processes for the success of infection, and (ii) regulation of responses against host barriers to infection. Most of them have been described in bacteria causing diseases concerning animals, especially in mammals and mostly humans.

### Regulation of Biological Processes

Key biological processes set the basis for the adaptation to the host molecular environment, including important responses such as: temperature sensing, stringent microbial response, biofilm formation and Quorum Sensing (QS), or regulation of virulence.

#### Temperature Response

One of the first cues helping the pathogen to perceive when it has entered the host is the change in temperature. Besides, it cannot be overlooked that a characteristic of infectious diseases is the hyperthermia response, which elevate the body temperature during inflammation (Kluger et al., 1998; Casadevall, 2016). While it is difficult to ascertain, it has been appointed that fever has beneficial effects for the protection against pathogens (Mackowiak, 1981; Casadevall, 2016), since such a high metabolic cost in higher vertebrates would have been lost during evolution if it did not present an advantage (Ostberg et al., 2000).

From a pathogen perspective, RNA presents advantages, as it has been shown to be a relevant molecular thermometer capable of controlling expression of heat shock and virulence genes (Narberhaus, 2010; Loh et al., 2018), when increasing temperatures melt secondary structures and allow access to the ribosome binding site (RBS) (Narberhaus et al., 2006). Through temperature monitorization pathogens can differentiate between free state, insect vector (if present), or hosts with regulated body temperature (González Plaza et al., 2016; Álvarez-Estrada et al., 2018), which could lead as well to develop a specific program to respond to fever (in hosts where this mechanism is present).

A recent study has evaluated the transcriptomic response of the psychrotrophic bacterium *Pseudoalteromonas fuliginea* BSW20308, which is adapted to Arctic environmental conditions (Liao et al., 2019). The aim was to evaluate the impact of global warming over the ecologically dominant genus, where temperature increases may trigger regulation mediated by sRNA. Authors described the whole sRNome (repertoire of sRNAs) when this microorganism grew at different temperatures, from very low ones resembling its natural environmental conditions, to higher temperatures of a global warming scenario. Results, according to authors, indicated an intense involvement of sRNAs in temperature adaptation. A 316 nt novel sRNA, termed Pf1, showed to be correlated with the expression to a wide group of 644 genes mostly annotated in the categories of catabolism, energy, translation, and intracellular transport. As previously mentioned, sRNAs have an ample range of effects over the transcriptome, helping bacteria to regulate their physiology upon environmental perturbations. Besides, since sRNAs are genetic carriers of information not translated into proteins, they can perform their regulatory roles in a faster fashion than other important regulators as heat-shock proteins. Although this characterization relies on an RNA-seq approach and further *in silico* data analyses, it is not surprising such a broad regulatory network as suggested in the article. Either by conservation or convergent evolution as appointed by Narberhaus (2010), this regulatory response is present in many pathogens for adaptation to the host conditions, and may be an important variable to consider during pyrexia (Mackowiak, 1981; Kluger et al., 1998; Ostberg et al., 2000; Casadevall, 2016; González Plaza et al., 2016).

In their analysis of the differential expression between infective and environmental temperatures in *Borrelia burgdorferi*, Popitsch et al. (2017) report a large set of sRNAs with differential expression between both conditions, and also reveal a variety of transcription origins. A brilliant conclusion of this study is that in overall, the mode of action of sRNAs could deeply impact the way we perform genetic studies. Deletion of a gene could erase as well an important regulator for a number of downstream targets. This can undoubtedly represent a confounding factor for the interpretation of results on loss-of-function phenotypes due to the interference of sRNA regulation.

**FIGURE 2:**
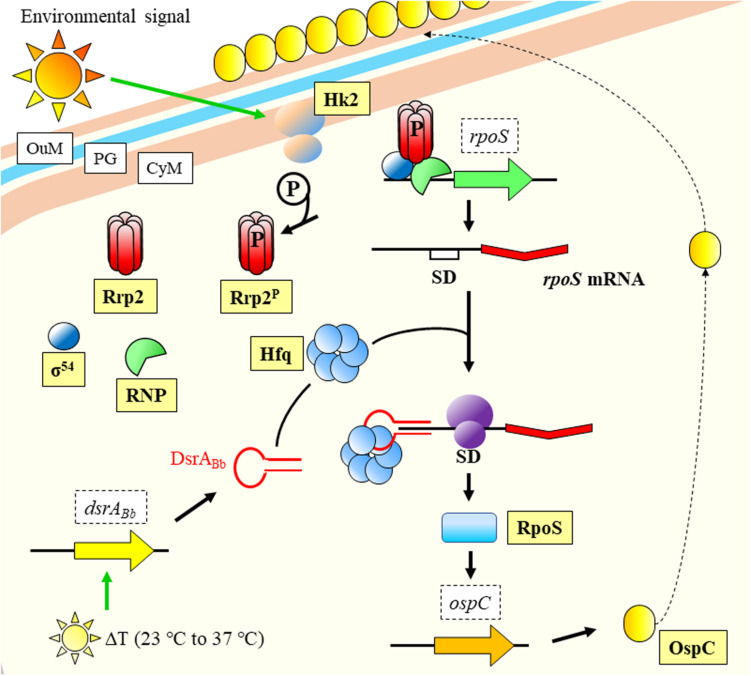
Temperature response model of *Borrelia burgdorferi*. An environmental signal activates Hk2 membrane protein that phosphorylates and activates Rrp2. Together with RNP and σ^54^, they facilitate transcription of *rpoS*. The mRNA is stabilized by the joint action of DsrA_Bb_ (expressed upon temperature increase to host conditions) and Hfq, and the messenger is translated into protein. RpoS regulates the transcription of *ospC*, which product is displayed on the OuM. CyM, Cytoplasmic membrane; OuM, Outer membrane; PG, peptidoglycan; RNP, RNA Polymerase; SD, Shine-Dalgarno. Black thick arrows indicate flow of a biological process, e.g., protein translation. Green thick arrows indicate activation. References: Burtnick et al., 2007; Lybecker and Samuels, 2007; Lybecker et al., 2010; Radolf et al., 2012; Steere et al., 2016.

What becomes clear is that monitorization of temperature changes is a central event in the life cycle of bacterial pathogens (Papenfort and Vogel, 2010), and that regulation follows an intricate pathway with several levels. In a fascinating example of interaction between kingdoms and pathogen manipulation, the spirochaetal outer surface (lipo)protein (Osp) C (OspC) from *Borrelia burgdorferi* binds to the SALP15 salivary protein belonging to the tick vector *Ixodes scapularis*. The pathogen uses the insect protein to succeed in the transmission to the mammalian host and its infection (Ramamoorthi et al., 2005). The expression of *ospC* is influenced by RpoS (Hübner et al., 2001), which back was suggested by Lybecker and Samuels (2007) to be regulated by a sRNA in response to temperature (Figure 2). RpoS is a key global regulator controlling virulence or response to general stresses in several pathogens (Fang et al., 1992; Suh et al., 1999; Dong and Schellhorn, 2010; Battesti et al., 2011). The regulation of temperature responses mediated by RpoS, is modulated by the sRNA DsrA_Bb_ in *B. burgdorferi*, which is expressed upon increase in temperature (Lybecker and Samuels, 2007). Few years later, it was experimentally confirmed to occur through Hfq mediation (Lybecker et al., 2010; Figure 2).

#### Stringent Response

Stringent response can be defined as the set of bacterial conserved mechanisms activated during nutritional environmental stresses (Poole, 2012; Drecktrah et al., 2018). It produces a general decrease on the expression of genes related with growth, involving synthesis of proteins or nucleic acids, and enhanced transcriptional levels of genes for survival (Chatterji and Ojha, 2001; Poole, 2012; Irving and Corrigan, 2018). When the bacterial stringent response is unleashed, mediation of enzymes such as RelA or SpoT lead to global transcriptomic changes (Atkinson et al., 2011; Shyp et al., 2012). A different version of RelA in *Borrellia burgdorferi*, Rel_Bbu_, has regulatory capabilities on a third of the sRNAs identified in this bacteria to date (Drecktrah et al., 2018). This pathogen has a life cycle that includes a vertebrate and an invertebrate host, thus, adaptation to different environments in order to regulate the behavior in such disparate conditions represent a challenge for survival. Not surprisingly Drecktrah et al. (2018) found that most of the targets of the regulated sRNAs are involved in two biological processes required during conditions of infection, virulence and metabolism. Interestingly, the sRNAs targets of Rel_Bbu_ are not exclusively found within the chromosome, but could be found as well in plasmids.

#### Biofilm Formation and Quorum Sensing

**FIGURE 3:**
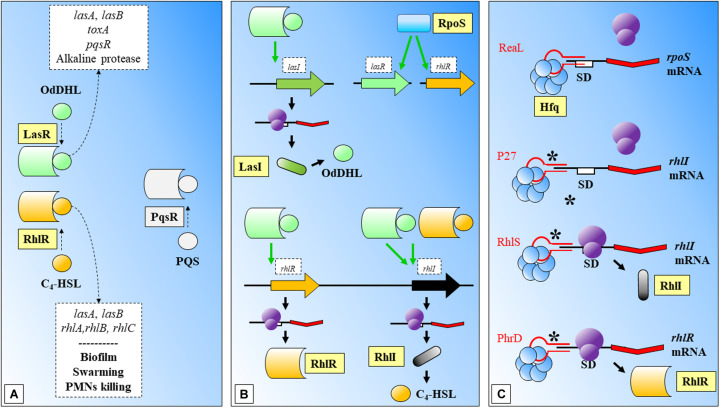
Biofilm formation and Quorum Sensing in *Pseudomonas aeruginosa*. **(A)** LasR and RhlR are two key regulatory molecules that need OdDHL and C_4_-HSL participation. LasR-OdDHL regulate the genes included in the upper box. RhlR-(C_4_-HSL) regulate genes or affect processes indicated in the lower box. PqrS is another important regulator, it has been shaded as it does not participate in the shown processes. **(B)** LasR and RhlR expression is dependent on RpoS. LasR-OdDHL regulates positively: the expression of *lasI*, and ultimately the synthesis of OdDHL; the expression of *rhlR*; and *rhlI*. RhlR-(C_4_-HSL) regulates positively the expression of *rhlI*. **(C)** Mechanisms of action of several sRNAs involved in regulation of the synthesis of regulatory proteins within these signaling systems: ReaL affects negatively the expression of *rpoS*; P27 is another negative regulator, in this case of *rhlI*; RhlS regulates positively *rhlI*; PhrD is a positive regulator of *rhlR*. C_4_-HSL: N-butanoyl-homoserine lactone; OdDHL: N-(3-oxododecanoyl)-L-homoserine lactone; PQS: Pseudomonas quinolone signal; SD: Shine-Dalgarno. Black thick arrows indicate flow of a biological process, e.g., protein translation. Green thick arrows indicate activation. Dashed-line text boxes: indicate downstream effects of the two signaling systems, phenotypes are indicated in bold (e.g., swarming). ^∗^: indicates that this mechanism of sRNA regulation has been proposed. References: Schuster et al., 2004; Nadal Jimenez et al., 2012; Brouwer et al., 2014; Pita et al., 2018; Thi Bach Nguyen et al., 2018; Chen et al., 2019; Malgaonkar and Nair, 2019.

Biofilms are bacterial community structures that provide additional protection against the host immune system, e.g., the effect of cytokines (Leid et al., 2005). Biofilm formation requires coordination of QS mechanisms of communication, which are mediated mainly by N-acyl homoserine lactones (AHLs) among other molecules and mainly controlled by the *rhlR*-*rhlI* and *lasR*-*lasI* signaling systems (Davies et al., 1998; Lee and Zhang, 2015; Figure 3). The AHL N-butanoyl-homoserine lactone (C_4_-HSL) is an important molecule in the QS response of *Pseudomonas aeruginosa*. This molecule binds to RhlR and the activated complex regulates positively the expression of *rhlI*, and its translation to RhlI that synthesizes C_4_-HSL. The synthesis of RhlR has been positively related with a 74-nucleotide sRNA, PhrD (Malgaonkar and Nair, 2019; Figure 3). The interaction between the regulator molecule and the mRNA target was predicted *in silico* and further demonstrated in experimental conditions (which mimicked the host during pathogenesis). While this system seems to act independently of any *P. aeruginosa* proteins (authors expressed it heterologously in *E. coli*), the system achieves better expression levels in the *P. aeruginosa* background, probably assisted by native proteins.

The involvement of sRNAs in the QS response of *P. aeruginosa* was investigated with mutants of AHL synthesis by Thomason et al. (2019), (Figure 3). Authors found a group of sRNAs responsive to AHLs treatment, where RhlS (previously SPA0104) (Ferrara et al., 2012) showed the highest accumulation at inductive conditions. This regulator acts positively over the translation of *rhlI*, leading ultimately to the production of C_4_-HSL. P27 is another sRNA that regulates negatively the translation of *rhlI* bindings to the 5′ UTR aided by Hfq (Chen et al., 2019; Figure 3).

The environmental bacteria *Burkholderia cenocepacia* belongs to the *Burkholderia cepacia* complex (Bcc) and can become an opportunistic pathogen in plants (Mahenthiralingam et al., 2005), but also in patients affected by cystic fibrosis (Drevinek and Mahenthiralingam, 2010). The sRNA ncS35 is involved in growth regulation by predicted binding to mRNA targets, probably facilitating the degradation of the transcript (Kiekens et al., 2018). Authors used a deletion mutant (*ΔncS35*) in comparison with the wild type (WT) strain, and a complemented *ΔncS35* overexpressing the sRNA under inductive conditions. The mutant phenotype displayed biofilms with larger aggregates, increased optical density or metabolic activity in comparison with the WT or the complemented mutant. Differential gene expression between the mutant and the WT showed upregulation of genes involved in metabolism both in exponential and stationary growth phases. This indicates a negative regulatory role of ncS35 over bacterial growth. Additionally, authors observed higher expression values for ncS35 when bacteria form biofilms than in free planktonic culture. Additional increases in transcription occurred during nutrient limitation after cultivation in M9 minimal media or in the presence SDS, a known membrane stressor (Flahaut et al., 1996). In overall, the effect of this sRNA is to slow-down growth. The higher expression during presence of stressors, may indicate its involvement in protection of the bacteria by restricting division when environmental conditions are detrimental for the pathogen.

#### Virulence

Bacterial virulence can be defined as the “relative capacity to overcome available defenses” (Sparling, 1983), or “the relative capacity of a microorganism to cause damage in a host” (Casadevall and Pirofski, 2003). This capability is mediated by virulence genes, which have to fulfill three requirements: (i) active in the interaction between pathogen and host, (ii) direct determinants of the pathogen damage, and (iii) the lack of those virulence genes in non-pathogenic strains (Wassenaar and Gaastra, 2001). Some authors also use the term “virulence factor” instead of “virulence gene” (Diard and Hardt, 2017).

**FIGURE 4:**
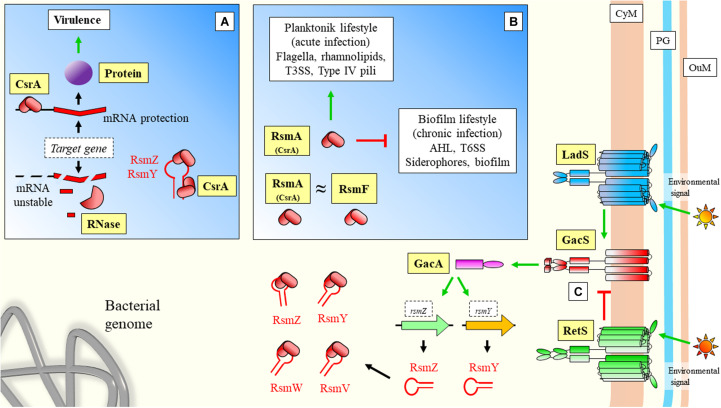
Virulence sRNA mediated regulation in *Pseudomonas aeruginosa*: Role of CsrA regulatory protein and sequestration by sRNAs. **(A)** CsrA has been described to have also a positive effect on target mRNAs by protecting transcripts from degradation. **(B)** Regulatory effects of CsrA. In the case of positive CsrA regulation of targets, when sRNAs bind and sequester this protein, they force the instability and degradation of the messenger **(A)**. In the case of CsrA negative regulation of targets (Figure 1A), sRNAs can prevent binding of the regulatory protein to the mRNA allowing translation (where sRNAs have a positive effect over mRNA targets). **(C)** GacS/GacA two component system is either activated by LadS, or inhibited by RetS. These two transmembrane proteins mediate between GacS and environmental stimulation. Upon activaction, Gac promotes the transcription of sRNAs, which can further bind CsrA/RsmA and modify expression of downstream genes. CyM, Cytoplasmic membrane; PG, peptidoglycan; OuM, Outer membrane. Black thick arrows indicate flow of a biological process, e.g., protein translation. Green thick arrows indicate activation. Red thick arrows with flat cap indicate inhibition. Text boxes: indicate downstream effects of RsmA. References: Wei et al., 2001; Records and Gross, 2010; Sonnleitner et al., 2011; Coggan and Wolfgang, 2012; Nadal Jimenez et al., 2012; Marden et al., 2013; Yakhnin et al., 2013; Chambonnier et al., 2016; Janssen et al., 2018a; Romeo and Babitzke, 2018; Valentini et al., 2018.

The role of sRNAs over virulence has been well-characterized in *Pseudomonas aeruginosa*. In this organism, an important system for regulation of virulence is the carbon store regulator (Csr) or repressor of stationary-phase metabolites (Rsm), being the main regulatory protein CsrA (or RsmA) (Figure 4). This system can also control other important features of the interaction with the host, such as biofilm formation, carbon metabolism, or stress responses (Romeo and Babitzke, 2018). Central to this system is the Gac/Rsm pathway, where the GacS/GacA two component system has a fundamental role (Coggan and Wolfgang, 2012; Figure 4). GacA induces the expression of the sRNAs *rsmY* and *rsmZ*, which can bind to the central regulatory protein RsmA (CsrA) to block its regulatory functions (Kay et al., 2006; Figure 4). RsmW is another sRNA that can bind to the regulatory protein (Miller et al., 2016; Valentini et al., 2018). This sRNA mediation can allow cells to respond precisely to environmental challenges, and aid transition from different infective phenotypes in *P. aeruginosa*. Additionally, RsmF/RsmN (a CsrA family protein), have overlapping functions to RsmA (Marden et al., 2013; Romero et al., 2018). Janssen et al. (2018a) have identified RsmV, a new 192-nt small non-coding RNA that has binding activity to RsmA and RsmF. *In vitro* electrophoretic assays confirmed that both proteins bind RsmV probe with high affinity. This interaction was supported by complementation studies using a two-plasmid reporter system in a mutant with high levels of RsmA/RsmF (lacking *rsmV*, *rsmY*, and *rsmZ*). Complementation with a plasmid expressing RsmV antagonized the activity of RsmA/RsmF (Figure 4). The displayed redundancy of targets with previously known sRNAs illustrates the fine-tuned coordination of sRNA regulation.

The sigma factor RpoS controls as well a wide number of virulence related genes in *Pseudomonas aeruginosa* under environmental stresses. RpoS translation has been shown to be negatively regulated by the sRNA ReaL, through a Hfq dependent base pairing mechanism (Thi Bach Nguyen et al., 2018; Figure 3). As authors point out, this was the first case of a negative sRNA transcriptional regulator of *rpoS*.

### Host Barriers to Infectious Diseases: sRNA-Mediated Bacterial Adaptation to a Hostile Environment

The second category groups those responses from the pathogen to known barriers of infection, such as acidic pH, inflammation, or nutritional immunity.

#### Acidic pH: One of the First Barriers to Infection

The acidic pH of the stomach represents one of the first barriers to infection set by the host. Microbial parasites can overcome it through different mechanisms, including the degradation of urea into CO_2_ and NH_3_ (Burne and Chen, 2000). This strategy increases the survival chances of enteropathogenic organisms, such as *Yersinia pseudotuberculosis*, a Gram-negative food-borne bacterial pathogen (Hu et al., 2010). Another mechanism involves modifying the composition of the cell membrane to withstand sudden exposure to acid pH, by incorporation of different membrane proteins, or the enzymatic modification of the pre-existing fatty acids already in the membrane (Bianco et al., 2019). Cell membrane composition changes have key roles during infective processes, as it determines the fluidity of toxic compounds or antimicrobials. The enzyme cyclopropane fatty acid synthase mediates the incorporation of a methylene group into unsaturated fatty acids. The enzyme transcripts were shown to be stabilized and protected from the degradative activity of RNAse E in *Escherichia coli*, by two sRNAs, RydC, and ArrS; while a third one, CpxQ, had a repressive role (Bianco et al., 2019). While RydC and ArrS mask the mRNA cleavage site that is not available to RNase E, CpxQ increases the accessibility to the same site.

#### Inflammation: Oxidative Stresses

When the host detect the presence of a pathogen, one of the characteristic responses is inflammation and the subsequent presence of oxidative stresses for the bacteria (Carlos et al., 2018). These stresses are common for extremophiles, such as the haloarchaeon *Haloferax volcanii*. A recent study reports the presence of hundreds of sRNAs in response to oxidative stress caused by hydrogen peroxide in this species (Gelsinger and DiRuggiero, 2018). Despite the potential evolutionary lineage distance between archaea and bacteria, this study shows as well how small regulatory RNAs can play a key role for regulation of biological processes in extreme conditions. In the case of infectious diseases, the extreme transient conditions relate to the stress caused by the host during oxidative burst, which induce heavily the expression of RsaC sRNA in *Staphylococcus aureus* (Lalaouna et al., 2019). The sRNA binds to the start codon region of the *sodA* mRNA, involved in protection against reactive oxygen species (ROS). The repression of the targeted gene allows the transcription of a second enzyme, SodM, involved in ROS protection but using iron as cofactor, instead of manganese (limited due to the nutritional immunity).

#### Nutritional Immunity

Besides oxidative stress (although related), pathogen recognition triggers other quick immune responses from the host, aimed to clear the bacteria by restriction of the available metabolic resources. The term “nutritional immunity” (Damo et al., 2013) refers to the limitation by the host of essential elements for the development of the pathogen. This strategy includes targeting of iron, manganese, or glucose (Carlos et al., 2018). Undoubtedly, the decrease in nutrients cellular levels has a direct relationship with the triggering of stringent mechanisms in bacteria. Regarding glucose metabolism, a characteristic behavior of human patients during infective diseases are patterns of transient anorexia that lead to a lower energetic intake, thus, limiting the availability of nutrients. This is a probable evolutionary response from the host, in order to create a metabolically stressful environment for the pathogen. Given that these host-pathogen relationships have developed through evolution, additional layers of regulation must have appeared in key microbial processes, in order to ensure survival of the bacterial strains, where sRNAs play a fundamental role.

In the case of manganese, the host immune system can limit its extracellular levels (Diaz-Ochoa et al., 2014), causing an impairment in the oxidative stress protection machinery from the pathogen, while increasing the oxidative burst. In that regard, the previous example of RsaC, can help *S. aureus* to avoid the synthesis of a non-functional enzyme (SodA) that requires manganese, and synthesize the second enzyme (SodM) that uses available iron as cofactor restoring the oxidative protection.

Besides targeting manganese, iron starvation is another innate immunity strategy of vertebrates, when detrimental bacteria are identified: the so-called “iron withholding strategy” (Ong et al., 2006). From a broader perspective, mechanisms to deal with iron deprivation have been described in environmental bacteria. For instance, the sRNA iron-stress activated RNA 1 (IsaR1) mediates the acclimation to conditions of iron starvation and high salinity in cyanobacteria (Rübsam et al., 2018). Even an additional role has been reported in the regulation of osmotic response. IsaR1 down-regulates the expression of the gene *ggpS*, encoding for the enzyme GG-phosphate synthase which is involved in the accumulation of heteroside glucosylglycerol (GG), and the adaptation to high saline concentrations. IsaR1 interferes with the 5′UTR of its target gene, *ggpS*. As it is becoming clear in the sRNAs research field, the *ggpS* regulation strategy does not follow an abrupt “all or nothing” scheme. It rather serves to integrate a wide range of environmental fluctuations in a continuous manner.

**FIGURE 5:**
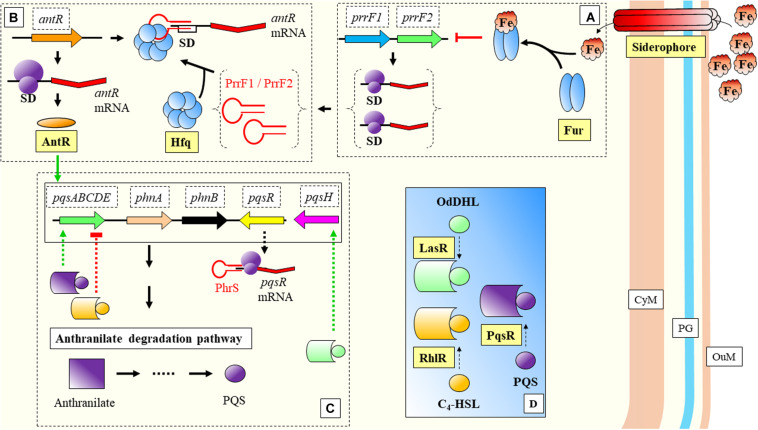
Nutritional immunity response in *Pseudomonas aeruginosa*. **(A)** Excess of extracellular iron is bound to Fur protein that inhibits transcription of PrrF1 and PrrF2. **(B)** PrrF1 and PrfF2 bind to the SD site by mediation of Hfq and block the translation of antR. **(C)** AntR has a positive effect over anthranilate degradation genes. Another sRNA, PhrS, allows the transcription of *pqsR*. **(D)** PqsR is an important regulator, which together with RhlR and LasR have key regulatory effects over genes belonging to the anthranilate degradation pathway (shown in panel **C**). CyM, Cytoplasmic membrane, OuM, Outer membrane; PG, peptidoglycan. Black thick arrows indicate flow of a biological process, e.g., protein translation. Green thick arrows indicate activation (equal for green thick dashed lines). Red thick arrows with flat cap indicate inhibition (equal for red thick dashed lines). References: Dubern and Diggle, 2008; Brouwer et al., 2014; Baker et al., 2017; Djapgne et al., 2018.

**FIGURE 6:**
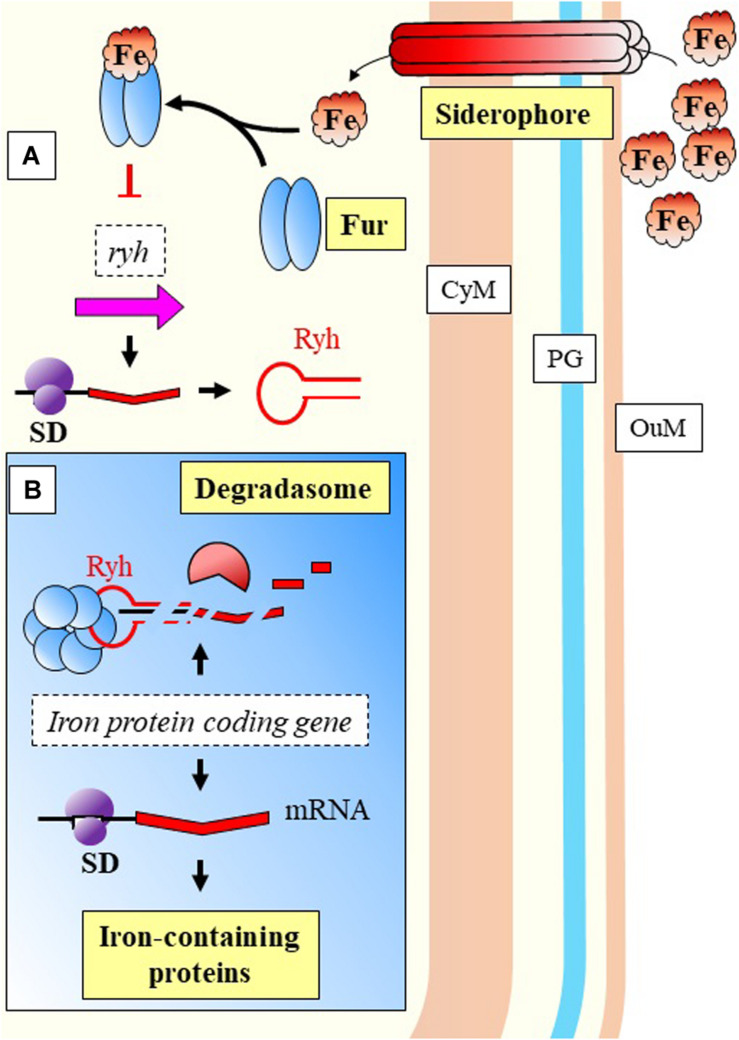
Nutritional immunity response in *E. coli*. **(A)** Excess of extracellular iron is bound to Fur protein that inhibits transcription of Ryh. **(B)** Ryh can inhibit the synthesis of proteins containing iron when this element is limited due to nutritional starvation exerted by the host. Ryh negative regulation is mediated by Hfq, binding to the target mRNAs directing the activity of the degradasome. CyM, Cytoplasmic membrane, OuM, Outer membrane; PG, peptidoglycan. Black thick arrows indicate flow of a biological process, e.g., protein translation. Red thick arrows with flat cap indicate inhibition (equal for red thick dashed lines).

The previous model of cyanobacterial regulation in response to low iron, is relevant as well during infection of *P. aeruginosa*. This bacterium is able to detect environmental iron fluctuations, which drive to the expression of virulence genes in response to the host primary line of defense. Two of its sRNAs, PrrF1, and PrrF2, react to iron (Fur mediated) and have been shown to repress anthranilate metabolism (Djapgne et al., 2018; Figure 5). These two sRNAs have been described as functional homologs of the RyhB in *E. coli* (Wilderman et al., 2004). The regulatory effect is indirect, as they inhibit the translation of a transcriptional activator, *antR*, with downstream effects over genes for degradation of anthranilate. The metabolic pathway from anthranilate degradation ends with the synthesis of *Pseudomonas* quinolone signal (PQS), relevant for Quorum Sensing (QS) (Brouwer et al., 2014; Figure 5). RyhB, was previously described as a negative regulator of genes involved in the control of iron levels within the cell (Massé and Gottesman, 2002). Besides the regulatory activities toward genes related with iron homeostasis, Massé and Gottesman reported the RyhB mediated regulation of three enzymes of the tricarboxylic-acid (TCA) cycle (succinate dehydrogenase, aconitase, and fumarase) (Figure 6).

Recently, the contribution of RyhB over the carbon metabolism was quantified (Lyu Y et al., 2019). A mutant was generated by recombination-mediated deletion of RyhB, in comparison with the WT and an inducible mutant. Authors report a redirection of the metabolic flux toward the pentose phosphate pathway. These results support the role of sRNAs in modification of central catabolism in order to adapt to changing environments and allow survival. According to authors, it may be due to the fact that key cellular catabolism enzymes have iron as component of their structure. Several of the enzymes involved in the TCA cycle require iron on their structure (Cornelis et al., 2011). The relationship between carbon and iron levels has been previously addressed in environmental bacteria (Kirchman et al., 2000), and similar mechanisms occur with pathogenic regimes (Andrews et al., 2003). Central carbon metabolism sRNA regulation has been also reported for *Escherichia coli*, according to environmental conditions (Shimizu, 2013; Lyu Y et al., 2019). That is of especial relevance during nutrient starvation, where alternative metabolic resources must be sought.

**FIGURE 7:**
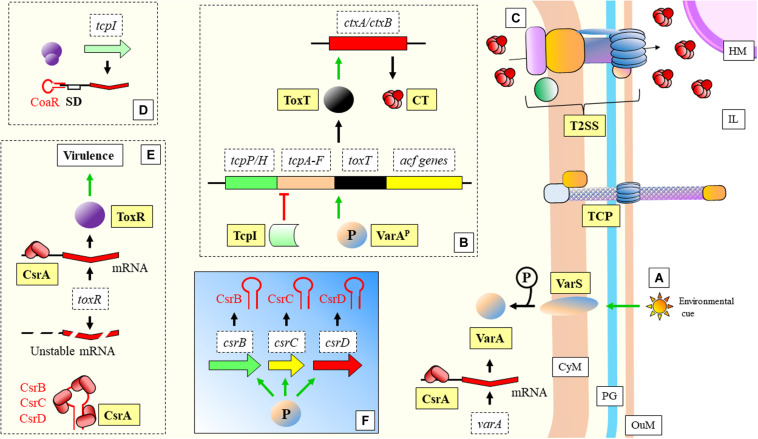
*Vibrio cholerae*: mechanisms of virulence and regulation. **(A)** Environmental cues trigger the signalization in the two-component system VarS/VarA (functional homologs to the GacS/GacA two-component system of *P. aeruginosa*). Phosporilation of VarA activates this protein. Transcription of *varA* is facilitaded by CsrA. **(B)** VarA^*P*^ (activated) acts as a transcriptional activator of *toxT*. ToxT activates as well *ctxA/ctxB* encoding the two subunits of the CT protein, which is extruded to the IL through a T2SS **(C)**. The *tcpA* gene, required for the assembly of the TCP, is located in the same operon as *toxT*. TcpI is a transcriptional inhibitor of *tcpA*. TCP structure is necessary for the progression of the disease (bacterial aggregation and microcolony formation). **(D)** CoaR sRNA blocks binding of the ribosome to the *tcpI* mRNA. **(E)** CsrA stabilizes *toxR* mRNA, which is necessary for positive regulation of virulence genes. **(F)** VarA^*P*^ activates transcription of CsrB, CsrC, and CsrC sRNAs, which sequester CsrA, yielding an unstable *toxR* transcript. CT, Cholera toxin; CyM, Cytoplasmic membrane; HM, Host cell membrane; IL, Gastrointestinal lumen; OuM, Outer membrane; PG, peptidoglycan; T2SS, Type II secretion system; TCP, Toxin coregulated pilus. Black thick arrows indicate flow of a biological process, e.g., protein translation. Green thick arrows indicate activation. Red thick arrows with flat cap indicate inhibition. References: Hammer and Bassler, 2007; Jang et al., 2011; Cobaxin et al., 2014; Mey et al., 2015; Ng et al., 2016; Dorman and Dorman, 2018; Jemielita et al., 2018; Butz et al., 2019; Xi et al., 2020.

Another example of adaptation during nutrient limitation can be found in the Gram-negative bacteria *Vibrio cholerae*, responsible for cholera disease. A set of sRNAs (CsrB, CsrC, and CsrD) (functional homologs of RsmA protein and RsmB/C/D sRNAs in *P. aeruginosa*) bind to this protein through a region that resembles the SD of the CsrA mRNA targets (Butz et al., 2019; Figure 7). This bacterium adapts to the clear differences and limitations innutrient content between the human host and the external environment. The mannitol operon is related to the adaptation to aquatic environment and to biofilmformation, and is regulated by the non-coding 120 nt RNA MtlS (Mustachio et al., 2012; Zhang and Liu, 2019). Zhang and Liu (2019) have shown what are the causes determining MtlSregulation. Very interestingly, it is not directly the environmental cue that triggers alterations in the expression level of the sRNA, but the mRNA levels of thetarget gene, *mtlA*. Another sRNA recently described, CoaR, binds to the mRNA of *tcpI* to block its translation (Xi et al., 2020; Figure 7). TcpI is a negative regulator of *tcpA*, a gene encoding the structural major pilin subunit of TCP (Harkey et al., 1994).

## Infectious Diseases in Plants

Most of the studies involving plants have been conducted in relevant crop species. Understanding the role of sRNAs has interest for breeders and companies, because advances in regulation of the disease could allow to control bacterial pathogens and prevent economic losses. In a world with continuous population growth, the improvement in agriculture efficiency among other measures could help to avoid shortages in food supply and help to mitigate the carbon footprint of agricultural practices (Beebe et al., 2013). Additionally, higher efficiency means that less agricultural land is required, and bigger efforts and extensions could be directed toward maintaining biodiversity (Trewavas, 2001). One of the most interesting aspects of the war between plants and bacteria, is the sessile nature of plants while bacteria have the advantage of being mobile. This fact has forced the evolutionary development in plants of sophisticated mechanisms of defense (Dangl and Jones, 2001). However, the immune response both from plants and animals, can cause a fitness disadvantage if it is held through time and not regulated. The eukaryotic sRNAs aid in the modulation of that response. But in the case of prokaryotes, sRNAs are relevant to fight back, involved in the regulation of different important pathogenic features during infection, such as secretion systems or mobility.

In this never-ending war biofilms help bacteria to endure different stresses, but plant immune systems have “learned” to target key molecules for its formation. Thus, mechanisms to evade the host recognition are necessary for the survival of the pathogen and establishment of a successful infection. In that regard, Nakatsu et al. (2019) have shown that not all isolates of *Pseudomonas syringae* produce AHLs. Many of the reported isolates carry mutations in two key genes: the AHL synthase *psyI*, or the AHL transcription factor *psyR*. Most probably the production of AHLs could have represented a biological burden for the infective processs. Strikingly, these strains still show responses of two sRNAs that should be related with AHLs, RsmY, and RsmX. Furthermore, their expression is enhanced at high cell densities, suggesting the existence of alternative routes for QS signaling. Although further research is needed, the loss of AHLs and the increased levels of these two sRNAs during situations with high number of cells, may reflect the involvement of these regulatory RNAs in mediating the coordination of a QS response.

In another brilliant event of the antagonism, plants have developed the capability to synthesize compounds that interfere with QS signalization. Rosmarinic acid (RA) was reported to bind with RhlR (instead of C_4_-HSL), inducing abnormal premature behaviors related with biofilm formation and virulence. When cultures of *Pseudomonas aeruginosa* PAO1 were challenged with RA, a group of sRNAs showed differential expression in response to the presence of RA, including the induction of RsmY (Fernández et al., 2018). This study highlights the relevance of sRNAs in the communication process between host and pathogen. If the host detects the presence of the bacteria, it can produce certain defensive compounds that will impair the virulence response of the pathogen through modification of important regulators and alteration of sRNA levels.

Plant-pathogen interaction it is not limited to detection and secretion, but it can reach up to a complex alteration of behaviors through genetic manipulation. Hijacking of the host metabolic machinery is not exclusive of viruses, it is a fine strategy of the gram-negative soil phytopathogen *Agrobacterium tumefaciens* (Gelvin, 2003; Hwang et al., 2017). The evolutionary counter-attack from plants has been the development of mechanisms to disrupt communication among pathogen cells, through the production of γ-aminobutyric acid (GABA) (Sheehan and Caswell, 2018). *Agrobacterium tumefaciens* and other Rhizobiales have an additional offensive strategy consisting in the production of AbcR1, a sRNA that regulates the plant transporter responsible for importing GABA molecules into the cell and its deleterious effects (Wilms et al., 2011). AbcR1 binds to the SD sequence and decreases the stability of the target mRNA.

A different sRNA, the highly conserved PmaR, can be found in *Agrobacterium* species and a specific strain of *Rhizobium* sp. It has been reported to be related to positive regulation of genes involved with peptidoglycan biosynthesis, motility, and virulence according to the study by Borgmann et al. (2018). Additionally, PmaR has a very important role in mediating ampicillin resistance, as observed after studying deletion mutants with impaired survival capabilities in growing concentrations of this antibiotic. The control of the antibiotic resistance gene was proposed by the authors as a means to obtain a biological advantage in the highly competitive rhizosphere environment. PmaR is a positive regulator binding to the 5′UTR, leading to the stabilization of the Shine-Dalgarno region, which would be otherwise prone to form structures preventing ribosome binding or even mRNAs destabilization.

Flowers are sensitive structures of plants, which can be used as an entry point by pathogens. Such an example is *Erwinia amylovora*, a Gram-negative bacteria responsible for fire blight disease in apple or pear trees belonging to Rosaceae (Oh and Beer, 2005). Once the bacterium enters the host, it spreads using flagella through the vascular system to continue infection. Three sRNAs dependent on Hfq (ArcZ, OmrAB, and RmaA) have been linked to the maintenance of the swimming and motility levels of this bacterium (Zeng and Sundin, 2014). However, the specific molecular regulation mechanisms were not understood. Given that motility is related with flagella, a potential target of regulation mediated by sRNAs is the dual system composed by the proteins FlhD and FlhC regulating the expression of the rest of the genes in the flagellar regulon (Liu and Matsumura, 1994; Frye et al., 2006). Schachterle et al. (2019) researched the involvement of ArcZ, OmrAB, and RmaA in flagellar regulation. First, they found reduced expression levels for regulatory and flagellar structural genes in lack-of-function mutants (Δ*arcZ*, Δ*hfq*, Δ*omrAB*, and Δ*rmaA*) in comparison with the WT. Besides, they observed similar *flhD* mRNA levels in double and triple deletion mutants compared to the single deletion mutants. While ArcZ and RmaA regulate the transcription of *flhD*, the same ArcZ and OmrAB affect post-transcriptionally *flhD* master regulator mRNA.

The broad host range plant pathogen *Pantoea ananatis* is responsible for yield losses in many important crops. The roles and targets of Hfq were unknown, and a recent study measured the molecular and phenotypical differences between a WT strain versus the *Δhfq* deletion and the *hfq* complementing mutant (Shin et al., 2019). The phenotype of *Δhfq* strains showed a range of phenotypical impairments such as slower growth rate, or loss of virulence when infecting onion. Within virulence traits, the mutants were affected in swimming motility, had a reduced AHL production, and a decreased ability to form biofilms. The complementing mutant resembled the WT strain phenotype. Authors identified the affected sRNAs by comparison of loss-of-function mutant with WT, both in low-density and high-density conditions. After data analysis, expression levels for 9 sRNAs were assayed through RT-qPCRs (*arc*Z, *fnr*S, *glm*Z, *rpr*A, *rye*B, *ryh*B2, *pPAR*237, *pPAR*238, and *pPAR*395), with reduced transcript levels for all them in the mutant. That was not the case for *glm*Z and *ryhB2*, and authors conclude that it is due to a negative regulation of those two targets by Hfq in WT strains of *P*. *ananatis*.

The study of Yuan et al. (2019) used *Dickeya dadantii* as a model bacterial pathogen, which depend on swimming motility for migration to the entry structures of the plant. RsmA (CsrA) and RsmB (CsrB) comprise a protein-sRNA system of regulators of the Type III Secretion System (T3SS) and other virulence phenotypes. While RsmA controls negatively *hrpL*, the master regulator of the T3SS, RsmB can sequester the protein. Besides RsmB, AcrZ (Hfq mediation) is as well involved in the regulation of motility and virulence in *D*. *dadantii*.

## Infectious Diseases in Animals

As previously stated, infection represents a competition relationship between pathogenic bacteria and a variety of hosts (ranging from plants to animals) for the same metabolic resources (Rohmer et al., 2011). The development of the field is much broader in humans due to the relevance of infectious diseases from an anthropogenic perspective. For other species, the research interest has been driven by economic interest in farming, because the studied species can potentially act as reservoirs for zoonoses (Slingenbergh et al., 2004), or their potential as model organisms.

### Non-vertebrates

Several non-vertebrate metazoans have attracted attention of the research community for different reasons, among them their similarity at innate immune responses with mammals (Tanji and Ip, 2005), because they constitute a vector for infectious diseases (Slingenbergh et al., 2004), or their importance as crop pests (Li et al., 2019).

The model organism *Caenorhabditis elegans* grazes on bacteria in the soil environment. Both bacteria and host establish a relationship which constitutes one of the most clear examples of interkingdom communication (Legüe and Calixto, 2019). It has been reported that *E. coli* sRNAs (OxyS and DsrA) can impact the expression of genes in the host (Liu et al., 2012). The uptake of non-self RNA molecules can happen via RNA transporters that have as well homologs in humans (Legüe and Calixto, 2019), or through membrane vesicle transport (Dauros-Singorenko et al., 2018).

Mutualistic endosymbionts are subjected to genome erosion, e.g., *Buchnera* in aphids (Wernegreen, 2002; Bennett and Moran, 2015). This reduction leads to losses in genes encoding for transcription factors, and highlights the importance of sRNAs as alternative regulatory elements. An early study by Hansen and Degnan (2014) found an interesting lack of mRNA expression in different life stages of *Buchnera*, while it was clear that proteins were differentially expressed. They predicted the involvement of a group of more than 600 sRNAs in protein regulation. Thairu et al. (2018) studied if these sRNAs were to be involved in post-transcriptional regulation events, by evaluation of two life stages of the endosymbiont, the extracellular proliferating stage in aphid embryos of *Acyrthosiphon pisum*, and the intracellular non-proliferating state in bacteriocytes of the same host. After RNA isolation (size ≤ 200 nt) and library preparation, authors performed sequencing, where data analysis provided a first indication of a group of 90 differentially expressed putative sRNAs. Authors evaluated *in vitro* the regulatory properties of one of these sRNAs, which putative target is *carB*, by cloning the regulatory element and its predicted coding sequence (CDS) target into two separate plasmids. The CDS was fused with the green fluorescent protein CDS (GFP), and indication in stabilization of mRNA would be suggested by enhanced fluorescence in the dual-plasmid system, in comparison with the control (where the vector that should contain the sRNA was empty). Results indicated that *carB* sRNA stabilizes the messenger of the target, and would compensate for the evolutionary loss of regulatory genes. But also, the changes in the insect diet can exert an effect over the endosymbiont. Thairu and Hansen (2019) measured through transcriptomic approaches if changes in the host diet would affect the sRNA regulatory pathways. Authors found that most of the potential targets among two conditions, when the aphid fed on two plants with notable differences in secondary metabolites, were related with amino-acid biosynthesis. The results are probably explained by the intimate relationship between the host insect and the endosymbiont, where changing in the host will drive a different transcriptional program to the cohabiting bacterium.

*Wolbachia pipientis* is an endosymbiont of insects that can affect the host behavior for ensuring its own vertical transmission (Werren et al., 2008). In experiments of infection of *Drosophila melanogaster* specimens or *Aedes albopictus* C6/36 cell lines with *W. pipientis*, Woolfit et al. (2015) examined the transcriptome response of both host and microorganism. Their results yielded a set of reads corresponding to intergenic regions of the bacteria that together with *in silico* candidates, allowed authors to identify two putative sRNAs. While further research is required for a better characterization of *Wolbachia* small regulatory RNAs, authors hypothesize that there could be an involvement in host manipulation.

### Vertebrates

High-throughput approaches evaluating the transcriptomic profile of both host and pathogen have become very important for the discovery of new sRNAs and their effect during the infection process (Saliba et al., 2017). The molecular behavior of pathogens is different when cultivated at *in vitro* conditions than when they are interacting with the host (Westermann et al., 2012). This method is known as “dual RNA-seq” (reviewed in Saliba et al., 2017; Westermann et al., 2017) and was used in bacterial infections models first by Westermann et al. (2016), studying the interaction between *Salmonella enterica* serovar Typhimurium and HeLa cells. Authors identified a highly expressed sRNA, PinT, which interacts with mRNAs by Hfq mediation. Not only this approach allows to characterize the bacterial sRNA profile, but also the study revealed a correlation of this sRNA with effects over the host immune pathways.

#### Non-human Models

A great fraction of the development on the field has been driven by economic interest, in order to understand the life cycle of causative agents for bacterial diseases in aquaculture farming industry. These settings concentrate in a short spatial range a significant number of specimens that could become up to 1,000 times higher than natural populations (Sundberg et al., 2016). Intensive fish farming has led to use of antibiotics, which together with the abnormal number of individuals, turn these exploitations into a hot-spot for the selection and spread of antibiotic resistance in the environment (McPhearson et al., 1991). Furthermore, the selection pressure within the microbiome due to interference and competition, directs toward quick changes in pathogen virulence that could be fixed in the genome (Sundberg et al., 2016). A necessary first step for an accurate understanding of the role of sRNAs at infection processes, is to survey the core repertoire of non-coding RNAs, as reported in a recent study (Segovia et al., 2018). Authors analyzed eleven *P. salmonis* genomes, having described more than 2,000 sRNAs (referred as non-coding RNAs, ncRNAs), from which more than 1,300 formed the ncRNA core group. Analyses of these RNAs have shown that many of the targeted genes in the bacterial genome show similarities to those described in section “sRNAs Regulate Key Processes for the Establishment of Infection.” of the current review. Among these similarities: manganese sensing response, membrane transport, components of the Type I and II Secretion Systems, expression of enzymes necessary for tissue colonization and acid resistance, or regulation of carbon flux.

Besides aquaculture, research in other vertebrates has been driven by biomedical interest in species that could serve as an alternative experimental model for human infectious diseases. In comparison with insect species, murine models show additional advantages, such as the presence of adaptive immunity or a wide range of available genetic resources. A murine macrophage line has been used to evaluate the interaction with *Brucella abortus*, a pathogen causing infectious diseases in cattle but also in humans (Golding et al., 2001; Budnick et al., 2018). Several sRNAs have been identified in *Brucella* spp., some of which have been associated with the pathogen virulence (Dong et al., 2018). When the sRNA BASI74 was overexpressed, the virulence of *Brucella* was negatively affected, while deletion had no effect. This is potentially explained by the presence of different copies of the sRNA in the bacterial genome, due to an important regulatory role. It means that while overexpression produces an excessive regulatory response, loss of an sRNA is compensated by different versions or alternative sRNAs. From an evolutionary perspective, it will help the pathogen to maintain regulation of important genes even when some sRNAs are lost from the genome.

#### Human as a Host

Recognition of bacterial infective agents relies on the innate immunity and a series of germline-encoded pattern-recognition receptors (PRRs) (Sellge and Kufer, 2015; González Plaza, 2018). Among others, TLRs strongly trigger systemic inflammation via macrophages and neutrophils (Moresco et al., 2011). These receptors can sense as well RNA molecules (Kawai and Akira, 2009), especially through TLR3, TLR7, and TLR8 (Hornung et al., 2008; Moresco et al., 2011). These receptors recognize molecules that are essential for the proper biological function of the bacteria, and thus, will be subjected to less evolutionary modifications than others (Akira et al., 2006).

But not in all cases bacterial RNA unleash an inflammatory response. Milillo et al. (2019) have reported down-modulation of MHC-II surface proteins in human monocytes/macrophages in the presence of *Brucella abortus* RNA. Because RNA is a molecule with a short lifespan and is subject to rapid degradation, it is present in alive bacteria rather than in dead cells. Authors argue that their findings suggest how *B. abortus* may thrive undetected within macrophages, due to the impairment of the MHC-II antigen presentation by inhibition of the gene expression. Nevertheless, the effect was also observed with partially degraded RNA. This molecular evasion could happen as well for many other pathogens, that could multiply and cause a serious disease requiring clinical treatment with antimicrobials. However, due to the current antibiotic crisis, the treatment of bacterial infections is becoming increasingly difficult due to the appearance of resistance (Bogaert et al., 2004). Given the role of sRNAs as regulatory molecules, it is clear that understanding their role during infection could lead to improved therapies to control the expression of resistance genes (Dersch et al., 2017). Focusing on sRNAs as therapeutic targets could be done in two main ways, targeting the mechanisms that regulate the virulence and infection related biological processes in the pathogen, or targeting the expression and regulation of antibiotic resistance genes. The latter group of genes are commonly shared among clinical pathogens in matter of years or months (O’Neill, 2014). Understanding interaction of one gene with its sRNA or set of sRNAs would likely be a universal therapy for different bacteria harboring the same resistance factor and become a promising research area for new treatments where bacteria will not presumably develop resistance (Ghaly and Gillings, 2018).

##### Respiratory diseases

One of the biggest issues during the onset of viral respiratory tract infections, is the frequent co-occurrence of bacterial infections that can dramatically aggravate the symptoms and the number of deaths (Mallia and Johnston, 2007). One of the explanations is that viral infections weaken host defense mechanisms, such as the clearance of bacteria by ciliated epithelial cells. Viral induced impairment of these processes create a favorable environment for the development of side bacterial infections by opportunistic pathogens (Hendaus et al., 2015).

Among those, *Staphylococcus aureus* can colonize different body surfaces and cause serious respiratory disorders (Lowy, 1998). The pool of more than 600 regulatory sRNAs present in this bacterium remains largely uncharacterized (Tomasini et al., 2014; Sassi et al., 2015; Carroll et al., 2016), but also provides an idea of the potential for adaptation to changing environments and success in the establishment of infection (Bayer et al., 1996). The global regulator *sarA* locus encodes a protein that regulate directly and indirectly genes involved in virulence (Dunman et al., 2001). This locus has three promoters (P2, P3, and P1 according to their genomic order) in a region of 850 bp upstream of the *sarA* coding sequence, and a 196 nucleotide sRNA (teg49) located within two of the promoters (Kim et al., 2014). Three overlapping transcripts including *sarA* ORF are produced. Mutagenesis assays in several regulatory sRNAs and the three promoters, indicated that teg49 is probably generated at the promoter P3 mRNA, likely through cleavage (Manna et al., 2018). Deletion of the P3 promoter resulted in the disappearance of both *sarA* P3 mRNA and teg49, besides lower SarA protein levels. Additional transcriptomics assays in the teg49 mutant, disclosed a group of genes which were up- and down-regulated in the absence of this sRNA. Those genes were involved in regulation, metabolism, and virulence. Teg41 is another sRNA involved in virulence regulation in *S. aureus*. It has been further characterized by Zapf et al. (2019; Table 2). This sRNA of approximately 200 nucleotides is located downstream of the transcript region of the potent phenol soluble modulin (PSMs) toxin type α. Authors propose that this sRNA positively regulates the toxin production by mRNA stabilization. Among different assays, they showed how the deletion of the 3′ sRNA region induced decreased levels of the αPSM transcripts, while higher levels of the toxin transcript were reported when Teg41 was overexpressed. The metabolic adaptation to nutritional starvation is also present in this pathogen, with the participation of sRNAs for the regulation of metabolism, as shown for RsaE (Bohn et al., 2010). A recent study on this regulator compared the transcriptomic response of two RsaE mutants, one by deletion, and the other by addition of an inducible promoter, with a wild type strain (Rochat et al., 2018). This sRNA has an effect over genes encoding enzymes of the TCA cycle, but also in the regulation of arginine catabolism, which was newly reported in this study.

**TABLE 2:** Relevant sRNAs of several infectious diseases having humans as a host.

**Pathogen**	**Infection**	**sRNA**	**Mechanism of action**	**Effect on bacterial physiology**	**References**
*Escherichia coli* O157:H7	Enterohemorrhagic	DicF	Hfq mediated. Liberates a secondary structure blocking the SD site of *pchA* mRNA.	Promotion of virulence: indirect enhancement of the expression of the LEE pathogenicity island during low oxygen conditions.	Melson and Kendall, 2019
*Escherichia coli* O157:H7	Enterohemorrhagic	Esr41	Hfq mediated. Forms a ternary complex with *ler* mRNA to repress *ler* expression.	Regulation of *ler* decreases adhesion ability of the pathogen, activation of *fliA* transcription has a positive regulatory effect over flagellum genes, and ultimately mobility.	Sudo et al., 2018
*Listeria monocytogenes*	Listeriosis	LhrC	Binding upstream from RBS, decrease mRNA stability.	Regulation of heme use and detoxification.	Ross et al., 2019
*Listeria monocytogenes*	Listeriosis	Ril47	Binding to SD, decrease mRNA stability.	Regulation of *ilvA* expression prevents isoleucine synthesis.	Marinho et al., 2019
*Mycobacterium tuberculosis*	Tuberculosis	6C	Negative regulation of targets by chaperone-independent binding to mRNAs.	Some targets include DNA replication or protein secretion.	Mai et al., 2019
*Mycobacterium tuberculosis*	Tuberculosis	189 sRNAs; MrsI	Target binding by non-canonical chaperones.	The studied sRNA is expressed during iron starvation and membrane stress.	Gerrick et al., 2018
*Mycobacterium tuberculosis*	Tuberculosis	ASdes	N/A	sRNA detected in plasma of patients, diagnostic biomarker potential.	Fu et al., 2018
*Salmonella enterica* serovar Typhimurium	Typhoid fever	STnc540	Hfq independent, mediation by ProQ.	Represses the expression of a magnesium-translocating P-type ATPase.	Westermann et al., 2019
*Staphylococcus aureus*	Opportunistic	Teg41	Suggested stabilization of the mRNA.	Positive regulation of PSM toxins.	Zapf et al., 2019
*Staphylococcus aureus*	Opportunistic	Teg49	Undetermined.	Teg49 potentially regulates regulatory factors, virulence, and metabolism. Together they affect virulence at infected tissues.	Manna et al., 2018
*Streptococcus pneumoniae*	Sepsis, meningitis, pneumonia	112 sRNAs	N/A	Regulation of different targets, some related to pathogen metabolism.	Sinha et al., 2018

*LEE, locus of enterocyte effacement; RBS, ribosome binding site; PSM, phenol soluble modulin.*

*Streptococcus pneumoniae* is a Gram-positive bacterium that causes a wide range of complications, including sepsis, meningitis, or pneumonia. It has been suggested that it was a magnifying cause of the high death rates during the 1918 influenza pandemic (Mallia and Johnston, 2007). Sinha et al. (2018) studied through a massive RNA sequencing *S. pneumoniae* strain D39W grown in laboratory conditions (Table 2). The first list yielded a total of 57 sRNAs, and authors raised the question if those were primarily expressed, or further cleaved by RNase to get to its final form. In that regard, differential RNAseq was carried out, and authors report a group of 44 novel sRNA candidates. These regulatory RNAs fall in three categories: antisense RNAs, short-antisense RNAs, or long-antisense RNAs. Some of these regulatory molecules have been proposed to regulate metabolic responses of the pathogen.

Tuberculosis is one of the most studied infectious diseases due to its incidence. It is estimated to affect 23% of the world population in its latent stage (Lyu L et al., 2019). Although previously the disease was projected to be eradicated by 2010, it has been continuously re-emerging due to a complex combination of factors (Cohen, 2000). The role of sRNAs has been long established, and there is a growing body of knowledge on the topic. However, as reported by Mai et al. (2019), there are considerable gaps of knowledge regarding small regulatory RNAs. The field has discovered until now a wide set of these molecules, but bigger efforts to elucidate their molecular mechanisms of action are needed. The sRNA 6C (six cytosine residues) was hypothesized, by the authors of this study, to play an important role in regulation of cell division of *Mycobacterium tuberculosis (M. tb)* (Mai et al., 2019). In their experiments, the authors used a vector to overexpress 6C sRNA of *M. tb*, and transformed *Mycobacterium smegmatis*. Analyses of expression through RNAseq indicate that the potential targets of 6C could be under negative regulation, through base pairing to the mRNA targets at the C-rich loops. While in Gram-negative bacteria the interaction must be mediated by Hfq, in high GC Gram-positive bacteria has been hypothesized the presence of putative chaperones, or as in this study by direct binding mechanisms independent of chaperones (Mai et al., 2019; Table 2).

Additional regulation of *Mycobacterium tuberculosis* by sRNAs was provided in the study of Gerrick et al. (2018), who tested the landscape of small regulatory RNAs in the presence of five stresses relevant for the pathogen, having found 189 sRNAs (Table 2). The direct interaction of one of those sRNAs, MrsI, with the target mRNA is potentially mediated by non-canonical chaperones, given that there are no described HfQ or ProQ homologs in mycobacteria. MrsI is expressed in conditions of iron starvation and membrane stress, when the host immune system triggers an inflammatory response including an iron withholding strategy, especially when entering macrophages. This response seems to be activated when the bacteria starts to suffer membrane and oxidative stresses, as the previous state of iron deprivation. This anticipatory response is not exclusive for *M. tuberculosis*, but according to authors has been observed in different prokaryotic organisms and it may have been developed as an evolutionary response to the inflammatory response by the host.

There is interest in developing sRNAs as stable markers for early diagnosis of the disease (Fu et al., 2018). Bacterial cell cultures of tuberculosis were studied by Fu and collaborators (Fu et al., 2018), who report four types of sRNAs (ASdes, ASpks, AS1726, and AS1890) (Table 2). On a first phase of this study, the authors tested whether these sRNAs would be detectable in the supernatant of cultures of *Mycobacterium bovis* BCG. Cultures of BCG were selected because the regulatory RNAs display 100% identity *in silico* between both strains. Once it was clear that the methodology was robust for identification of sRNAs from supernatant, authors tested the plasma of infected patients (*Mycobacterium tuberculosis*) in comparison with healthy controls. Among the four above mentioned regulatory RNAs, only ASdes was traced to the plasma of most tuberculosis patients, with statistical significance. These results are promising for the development of quick diagnostic assays based on the detection in blood or saliva of sRNAs derived from pathogens.

##### Gastrointestinal pathogenic diseases

**FIGURE 8:**
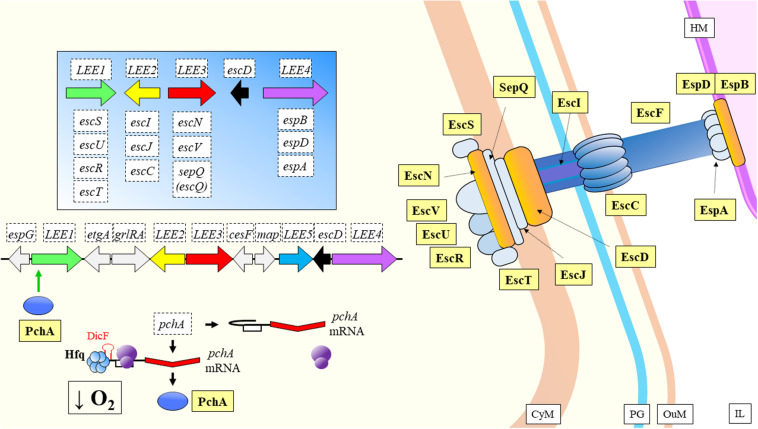
Type III secretion system in EHEC. The *pchA* transcript has autoinhibitory activity, as it forms a secondary structure covering the SD site. When concentration of oxygen is low, DicF by mediation of Hfq frees the SD of pchA, allowing translation. PchA activates transcription of LEE genes involved in the synthesis of components of the T3SS. CyM, Cytoplasmic membrane; HM, Host cell membrane; IL, Gastrointestinal lumen; OuM, Outer membrane; PG, peptidoglycan; T3SS, Type III secretion system. Black thick arrows indicate flow of a biological process, e.g., protein translation. Green thick arrows indicate activation. References: Deng et al., 2004; Pallen et al., 2005; Izoré et al., 2011; Ruano-Gallego et al., 2015; Furniss and Clements, 2018; Pena et al., 2019.

**FIGURE 9:**
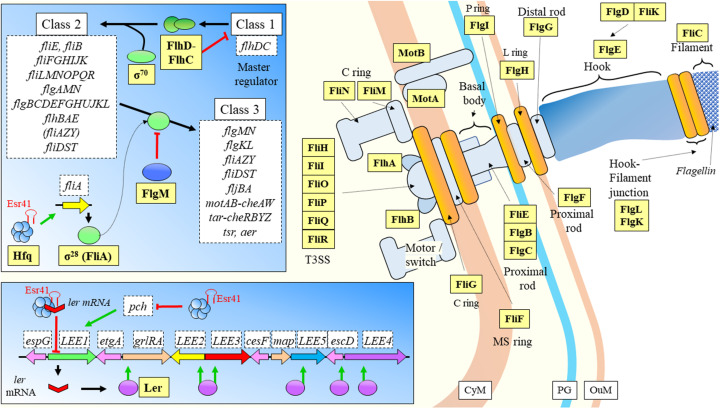
EHEC flagellar system. The *fliA* encodes for a transcription factor controlling expression of class 3 genes. The sRNA Esr41 regulates positively via Hfq mediation *fliA*. The flagellar system is regulated by sRNAs at a key point. Esr41 has as well repressing activities over LEE1 operon, when it forms a ternary complex with Hfq and *ler* mRNA. This inhibition compromises coding of Ler protein, which is a transcriptional activator of different genes at the LEE. Esr41 has as well inhibitory activities over *pch* that facilitate LEE1 gene expression through promoter binding. CyM, Cytoplasmic membrane; HM, Host cell membrane; IL, Gastrointestinal lumen; OuM, Outer membrane; PG, peptidoglycan; T3SS, Type III secretion system. Black thick arrows indicate flow of a biological process, e.g., protein translation. Green thick arrows indicate activation. Red thick arrows with flat cap indicate inhibition. References: Kalir et al., 2001; Chevance et al., 2006; Frye et al., 2006; Liu and Ochman, 2007; Chevance and Hughes, 2008; Sudo et al., 2014, 2018; Bhatt et al., 2016 and KEGG (https://www.genome.jp/kegg/pathway/eco/eco02040.html).

When enterohemorrhagic *Escherichia coli* O157:H7 (EHEC), a food-borne pathogen, enters the digestive tract, it must adapt from oxygen rich environments to an increasingly oxygen-limiting environment toward the colon, where pathogens sense the differential gas concentrations and start to express their virulence factors (Melson and Kendall, 2019). As mentioned above, this is a key behavior of bacterial strains in order to set the basis of the infection onset, which seems to have evolved to start from a very low number of input cells (Van Elsas et al., 2011). DicF is a sRNA dependent on Hfq directly targeting the SD sequence of the transcriptional activator *pchA*, to promote its expression under low oxygen conditions (Melson and Kendall, 2019; Table 2). Ultimately, DicF has an effect over virulence by indirect increase of the expression of the locus of enterocyte effacement (LEE) pathogenicity island, but only when Hfq is present and oxygen concentration is very low (Figure 8). This means that during the disease establishment and progression, the pathogen has a mechanism to sense when it has reached the colon so that the virulence factors should be expressed at the right time. One of the most relevant and novel aspects of the previous study, is the reporting on a sRNA that has a positive expression effect by liberating a transcriptional activator virulence gene from its own mRNA repressive activities (Figure 8). Another sRNA was recently shown to be involved in regulation of LEE and flagellar genes (Sudo et al., 2018). Esr41 requires Hfq mediation, and has regulatory functions over cell motility (Sudo et al., 2014) by induced activation of *fliA*, which encodes the transcription factor σ^28^ that regulates the expression of the class 3 flagellin subunit genes (Aldridge et al., 2006). Esr41 forms a ternary complex with the *ler* mRNA and Hfq for repression of the gene (Figure 9), leading to consequent repression of the LEE and a decrease in the adhesion ability of the pathogen (Sudo et al., 2018).

*Listeria monocytogenes* is the causative agent of listeriosis (Ágoston et al., 2009), characterized by different episodes of fever, diarrhea, or meningoencephalitis among other symptoms (Swaminathan and Gerner-Smidt, 2007). There are mainly two forms of this disease, the non-invasive gastrointestinal febrile manifestation, and the invasive life-threatening form causing for instance meningoencephalitis (Allerberger and Wagner, 2010). In this organism, the seven sRNAs comprising the LhrC family act by regulating the target *tcsA* that encodes a virulence protein, CD4^+^ T cell-stimulating antigen (Sanderson et al., 1995). The regulatory mechanism of LhrCs was recently elucidated, and follow a non-canonical pathway, where sRNAs bind far upstream of the RBS, impairing the stability of the mRNA through the participation of an unknown RNase decreasing the messenger half-life (Ross et al., 2019; Table 2). During the nutritional starvation confronted by the pathogen in the host, the bacteria can make use of the available iron at the host iron-containing proteins, as the heme group in hemoglobin (Cassat and Skaar, 2013). LhrC1-5, belonging to the LhrC family of sRNAs, has been related to the regulation of heme usage in this pathogen. dos Santos et al. (2018) have shown the involvement of these five sRNAs in the regulation of three genes that control heme toxicity (*tcsA*, *oppA*, and *lapB*). Besides, authors found indications of regulation through these sRNAs of three genes related with the uptake and use of heme. Host induced starvation of amino acids is one of the strategies of nutritional immunity used by mammalian hosts to limit the infection (Zhang and Rubin, 2013). In *L. monocytogenes* Ril47 is an sRNA under control of the stress activated sigma factor sigma B (σ^*B*^), which is involved in a plethora of environmental stresses affecting the pathogen (Dorey et al., 2019). Ril47 binds to the SD sequence of the *ilvA* mRNA decreasing its stability, as suggested by a recent study (Marinho et al., 2019). The consequence is a negative regulation of isoleucine biosynthesis by prevention of threonine deaminase synthesis (which is encoded by *ilvA*).

Bacteria belonging to the genus *Salmonella* cause mainly two affections: diarrheal disease and typhoid fever. The latter has a mortality rate of 10−20% if it is not treated with antibiotics, being its causative agent is *Salmonella enterica* serovar Typhimurium (Fàbrega and Vila, 2013). An example of influence of sRNAs over the nutritional adaptation of this pathogen was provided in the study of Kröger et al. (2018), where they report two novel sRNAs involved in positive (STnc1740) and negative (RssR) modulation of the growth of *S. enterica* serovar Typhimurium, through utilization of myo-inositol as carbon source. While the role of Hfq has been widely studied in this pathogen, Westermann et al. (2019) evaluated the contribution of ProQ as alternative RBP. Authors followed a previous study where ProQ was reported to display novel posttranscriptional regulatory activities (Smirnov et al., 2016). The study from Westermann sought to shed light over the role of ProQ in virulence by altering the phenotype (deletion of the RBP) and used a dual RNA-seq approach. The invasion capabilities of the single mutant were decreased (2-fold vs. WT), but no dramatic differences were found when the RBP was overexpressed. While these phenotypes were observed when infecting HeLa cells, very mild virulence was reported during infection of human phagocytic cells. Further evaluation of the dual RNA-seq results (HeLa cells as host) revealed alteration in metabolism of the host and immune signaling pathways. The mutant pathogen had a range of transcriptomic alterations over genes involved in chemotaxis and motility (downregulation), or in ribosomal and invasion genes (upregulation). In their analysis authors identified STnc540 as an sRNA independent of Hfq and regulated by ProQ. The interplay between sRNA and RBP was further shown to affect the expression of a magnesium-translocating P-type ATPase.

##### Antibiotic resistance

Antibiotic resistance is a serious concern for society that threatens health systems worldwide (O’Neill, 2014). One the biggest problems is the continuous appearance of novel mechanisms of resistance often fuelled by the natural presence of those mechanisms in the environment, quick global spread of antibiotic resistance genes (ARGs), uptake by pathogenic bacteria that can become resistant to several antibiotics, and lastly the lack of alternatives for treatment of infections caused by multidrug resistant bacteria (MDR) (O’Neill, 2014). The emergence of novel antibiotic resistance genes has dramatic consequences when they reach the clinical practice as they will be irreversibly fixed (Böhm et al., 2020). This brings to memory ideas of bacterial caused plagues in the pre-antibiotic era, when there were no means of controlling pathogens. Resistance to antimicrobials is additionally shaped by environmental stresses (in many cases of anthropogenic origin) faced by bacteria (Poole, 2012), but also interestingly coincide with those highlighted in section “sRNAs Regulate Key Processes for the Establishment of Infection.”

Genes conferring resistance to antibiotics are essentially subjected to genetic regulation, and thus, their expression can be as well tuned by sRNAs (Lalaouna et al., 2014). This fact makes sRNAs a potentially invaluable tool to treat emerging infections mediated by antibiotic resistant bacteria (ARBs). An increasing number of studies point toward that direction, and have been excellently reviewed by Lalaouna et al. (2014), Dersch et al. (2017), Felden and Cattoira (2018). The antibiotic susceptibility of multidrug-resistant (MDR) *Pseudomonas aeruginosa* was dramatically altered when AS1974 sRNA was overexpressed (Law et al., 2019). Authors of the study carried out a first screening on the sRNAs repertoire on three MDR clinical isolates, having found three small-regulatory RNAs that were significantly downregulated. Targets of AS1974 are involved mostly in pathways related with drug-resistance, but also regulation over oxidative stress, pilus biogenesis, and iron acquisition.

*Escherichia coli* can activate a stress-response mechanism mediated by RpoS (Poole, 2012). However, this mechanism is under regulation of a non-coding RNA, rprA, which induced ampicillin resistance when overexpressed (Sahni et al., 2019). The mRNA levels of this sRNA had been previously shown to increase in cells when treated with sub-inhibitory concentrations of ampicillin (Gutierrez et al., 2013).

Shigella strains are gaining attention due to the accumulation of resistances toward antibiotics (Puzari et al., 2018), for instance using efflux pumps to extrude fluoroquinolones from their intracellular space (Roy et al., 2020). Gan and Tan (2019) evaluated whether the sRNA SdsR, a transcriptomic regulator of the efflux pump *tolC* in *E. coli* and *Salmonella* (Choi et al., 2018), plays as well a regulatory role in *Shigella sonnei* or not. Although in previous reports SdsR lowers the levels of TolC mRNA and increases its sensitivity to fluoroquinolones, in their study the authors found that at decreasing levels of TolC mRNA the strain was less sensitive to antibiotics, such as norfloxacin.

## Discussion

This review focuses on the latest advances on small non-coding regulatory RNAs involved in bacterial infections, a field that has grown substantially in the last 2 years since the appearance of a previous review article (González Plaza, 2018). The importance of studying infectious diseases lies within the fact that global warming can lead to changing patterns in the distribution of bacteria causing diseases, or in the vectors harboring those microorganisms (Cohen, 2000; Epstein, 2001; Rodó et al., 2002; Khasnis and Nettleman, 2005). But not only from an anthropogenic perspective, these changing patterns in the distribution of infectious diseases can even cause the extinction of whole species (Harvell et al., 2002; Pounds et al., 2006).

Transference of information is a key process for quick adaptation to dynamic environments or species interactions, being one clear example the host-pathogen relationship. Infectious diseases have been shaped by co-evolution between pathogen and animal or plant hosts, both competing for the same metabolic resources. Due to the nature of RNA as a molecule with a short life-span, its role for modulation of environmental signals is of paramount importance for success of infection. This process represents a challenge for the survival of the bacteria, because the host sets a wide range of measures to limit the growth of the microbial strains, eliminate them, and recover homeostasis. The overall role of sRNAs is to fine-tune the bacterial response to different stresses, with an impressive amount of mechanisms of induction/repression of transcription. Not only they affect specific genes involved at synthesis/degradation pathways, but in many cases, they regulate the activity of transcription factors and regulators. Out of several studies it is clear that sRNAs can have multiple targets, and even achieve regulatory redundancy with other non-coding regulatory RNAs.

Innate immune system response can limit nutrient (e.g. iron and manganese) availability to induce death of the pathogen, and a series of sRNAs help bacteria to quickly adapt to immune system challenges, such as the iron or nutrient withholding strategy, where the host limits the available resources in order to limit the survival chances of the invading bacteria. Bacteria encounter some other challenges in the host, for instance acid pH in the stomach of animals, or inflammation leading to oxidative stress. Many of these microbial responsive strategies seem to be conserved from environmental bacteria to pathogens (e.g., involvement of sRNAs in oxidative stress response due to salinity or inflammation), although it could have appeared owing to convergent evolution since sRNAs are very flexible molecules in their activities.

As presented in this review, a very useful approach, is to study both mRNA and sRNAs in the same samples, because it can lead to elucidate regulatory networks.

A number of the studies reviewed here are focused on surveying the sRNome repertoire, or also on the regulation of targets. There is a need to carry on with these studies, since this basic research is fundamental for being able to understand e.g., which genes could be directly modulated by the pathogen. This brings to memory the Spanish Presidential Elections in 2019, where as always, political parties kept promising the rise in research funds to 2% of GDP. Although every Spanish Scientist understands the magnitude of this false assertion, it was concerning how politicians have made theirs the mantra of “Applied Science” as the sole generator of progress and development. For instance, Pfizer was reported to have abandoned recently their program of applied research in neurodegenerative diseases (Trullàs, 2019), which should be an example of “*how could we apply what we do not fully understand*?”. This seems to be a trend in every scientific system around the world (Lindsley, 2016), where the *application paragraph* is requested at every grant proposal, and does not seem possible to carry out a research project where the main aim is to obtain a “*general understanding of nature and its laws*” (Bush, 1945). It becomes clear why these type of basic studies are needed, because control of infections through drugs that can target pathogen sRNA regulation are not possible without a deep understanding of their structure, modes of regulation, behavior patterns during infection, or what genes alter their expression during infection. Small non-coding regulatory RNAs are a promising therapeutic target in the fight against multidrug-resistant bacteria, and since these molecules are less prone to changes, we may have a great opportunity to tackle antibiotic resistance through gene-based therapies.

Having said that, what is still largely missing in the field are more studies focusing on the inter-kingdom exchange of sRNAs, either as counterfeit to impair the activity of the bacteria, or as a pathogenic mechanism to hijack the metabolic machinery of the host. Many protein effectors, such as those translocated by the T3SS can modify the response from the host. In the case of sRNAs, membrane vesicles (MVs) can carry the genetic material to their targets protecting it from degradation (Toyofuku et al., 2019), and ultimately subvert the molecular behavior of the host (Ghosal, 2017; González Plaza, 2018).

Lastly, the wide regulatory effects of sRNAs over the transcriptome must be taken in account due to their potential to impact genetic studies where a loss-of-function phenotype is considered as suggested by Popitsch et al. (2017). Deletion may affect expression of unannotated sRNAs and have undesired effects over the phenotype because regulation of a number of targets could be lost. It could as well potentially affect the expression of targets if some coding genetic regions carrying sRNAs are overexpressed. These possibilities invite to consider carefully the presence of small regulatory RNAs in genetic studies, and maybe even revisit some of the studies to date.

## Author Contributions

JG reviewed the literature, constructed all tables and figures according to published literature, and wrote the manuscript.

## Conflict of Interest

The authors declare that the research was conducted in the absence of any commercial or financial relationships that could be construed as a potential conflict of interest.
